# Self-Assembled Nanogels Based on Ionic Gelation of Natural Polysaccharides for Drug Delivery

**DOI:** 10.3389/fbioe.2021.703559

**Published:** 2021-07-16

**Authors:** Huimin Wang, Hong Deng, Menghan Gao, Weiqi Zhang

**Affiliations:** State Key Laboratory of Medical Molecular Biology and Department of Biomedical Engineering, Institute of Basic Medical Sciences Chinese Academy of Medical Sciences, School of Basic Medicine, Peking Union Medical College, Beijing, China

**Keywords:** nanogel, drug delivery, polysaccharides, self-assembly, ionic gelation

## Abstract

The polysaccharides (PS) have been widely used as biomaterials in drug delivery, due to their excellent biocompatibility, ease of functionalization, and intrinsic biological activities. Among the various PS-based biomaterials, the self-assembled PS nanogels (NG) featuring facile preparation are attracting evergrowing interests in various biomedical applications. Specifically, NG derived from the self-assembly of natural PS well maintain both the physicochemical and biological properties of PS while avoiding the chemical modification or alteration of PS structure, representing a potent drug delivery system for various therapeutic agents. In this review, the natural PS, such as chitosan, alginate, and hyaluronan, for self-assembled NG construction and their advantages in the applications of drug delivery have been summarized. The residues, such as amine, carboxyl, and hydroxyl groups, on these PS provide multiple sites for both ionic cross-linking and metal coordination, which greatly contribute to the formation of self-assembled NG as well as the drug loading, thus enabling a wide biomedical application of PS NG, especially for drug delivery. Future developments and considerations in the clinical translation of these self-assembled PS NG have also been discussed.

## Introduction

Nanogels (NG) are nanoscale hydrogels that are composed of a three-dimensional network of polymers with the ability to adsorb a large amount of water. The NG has the merits of most of the nanoparticles (NP) for the applications of drug delivery, while it also possesses its own beneficial features due to its unique physiochemical properties (Soni et al., 2016; Xu et al., 2017). With a rational design, NG can have high hydrophilicity, tunable size and porosity, deformability, and degradability. The NG with an appropriate size (i.e., ~10–200 nm) can mediate the selective tumor accumulation for anticancer drug delivery by taking advantages of the enhanced permeabilization and retention (EPR) effect and the targeting ligands attached on the NG (Kobayashi et al., 2014; Hartshorn et al., 2018; Cuggino et al., 2019). The gelation of hydrophilic polymer generates NG, and meanwhile, the cross-linking degree controls the NG softness. The softness is a key parameter for the interaction of NP with the biological system (Li et al., 2020). For example, NG of different softness demonstrates a varied cellular uptake (Guo et al., 2018; Zhang et al., 2020). The deformation of NG allows it to pass through the pores that are smaller than the hydrodynamic size of NG, which plays an important role in the *in vivo* circulation and accumulation of NG at the disease site, e.g., tumor (Hendrickson and Lyon, 2010; Anselmo et al., 2015). The porous structure of NG provides large spaces for drug loading such as small-molecule drugs, nucleic acids, and proteins (Mauri et al., 2018; Suhail et al., 2019). The release speed of the drugs, a key factor for the drug delivery system, could be tuned by controlling the cross-linking degree of NG. More importantly, NG are generally hydrophilic and can be degraded when degradable or natural polymers are adopted as NG matrix. Among the naturally occurring polymers, polysaccharides (PS) represent one of the most prevalent biomacromolecules to construct NG for drug delivery.

Polysaccharides are biopolymers composed of repeated monosaccharides linked by glycosidic bonds. Different monosaccharides and their glycosidic linkage patterns generate various PS such as chitosan (CS), alginate (ALG), and hyaluronan (HA) (Liu et al., 2008). PS could be digested by various enzymes and recycled when used as biomaterials in the body. Most of the PS demonstrate low toxicity and immunogenicity, which make them appealing for various biomedical applications such as drug delivery (Barclay et al., 2019). In addition, some PS also demonstrate intrinsic biological activities. For example, HA could be specifically recognized by CD44, a receptor overexpressed on many cancer cells. Consequently, the HA-based nanocarriers could be used for CD44-targeted anticancer drug delivery (Spadea et al., 2019; Gao et al., 2021). The cross-linking of PS through chemical reactions, such as UV light radiation and glutaraldehyde treatment, can generate PS NG with good stability (Debele et al., 2016). The chemical modification of PS provides flexible options to construct NG and load cargoes; however, it may not only compromise the biocompatibility but also influence the biological activities of PS. A high degree of chemical modification could retard the degradation of HA and also compromise the CD44-targeted effects (Bhattacharya et al., 2017; Kim et al., 2019). Self-assembled NG based on natural PS would maintain their biological activities and meanwhile provide the physicochemical properties of NG for drug delivery. The functional groups of PS can also mediate different non-covalent interactions. Specifically, the hydroxyl group of PS can mediate the hydrogen bonding while the amine and carboxyl groups can take part in both electrostatic interaction and metal coordination (Giammanco et al., 2015; Debele et al., 2016; Kim et al., 2017). These non-covalent interactions can involve multiple monosaccharide units simultaneously to cause both intermolecular and intramolecular cross-linking of PS, inducing the formation of self-assembled NG. The preparation of self-assembled PS NG requires neither chemical reactions nor a complicated purification process, representing a facile and cost-effective fashion to construct nanocarriers for drug delivery. The common self-assembly of native PS is mainly mediated by the physical cross-linking either through ionic gelation or through metal coordination, which is reversible. Depending on the PS type, the resultant NG can further entrap various drugs through electrostatic interaction, hydrophobic interaction, and π-π stacking, thus helping solubilize the drugs as well as increase their bioavailability. Furthermore, the non-covalent interactions between the encapsulated cargoes and PS were also believed to stabilize the self-assembled NG (Zhang and Tung, 2017; Cai and Lapitsky, 2020). In this review, we mainly focused on the self-assembly of native PS, such as CS, ALG, and HA (Table 1), and the related NG for drug delivery. The characteristics of these PS and the representative strategies to prepare the self-assembled NG are summarized. Ionic interaction and metal coordination are the main routes employed to initiate the self-assembly of NG. Recent progress of the self-assembled PS NG for drug delivery is introduced with a focus on both the advantages and the challenges to realize the efficient drug delivery. Finally, a perspective for the future development of self-assembled PS NG in translational medicine has been discussed.

**Table 1:** Key features of polysaccharides discussed in this review.

**PS**	**Molecular structure**	**Sugar units**	**Representative biomedical applications**	**Biological activity(s) for drug delivery**
CS	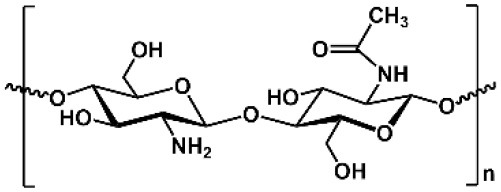	D-glucosamine, *N*-acetyl-D-glucosamine	Cosmetics, wound dressing, hemostatic agent, etc.	Encapsulation of anionic drug, mediating endosomal escape of drugs
ALG	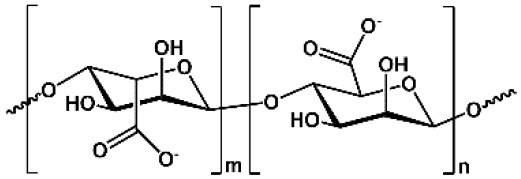	D-mannuronic acid, L-guluronic acid	Food additive, dental impressions, wound dressing, etc.	Encapsulation of cationic drugs
HA	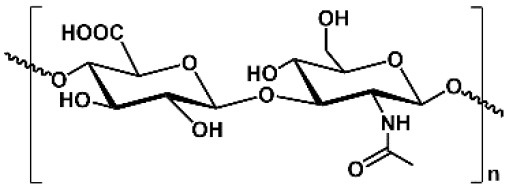	D-glucuronic acid, *N*-acetyl-D-glucosamine	Dermal fillers, joint lubrication, preventing postoperative adhesions, eye drops, etc.	Intrinsic targeting effect for HA receptors, e.g., CD44

*PS, polysaccharides; CS, chitosan; ALG, alginate; HA, hyaluronan*.

## Self-Assembled Chitosan (CS) NG

Among the self-assembled PS NG, CS is the most prevalent PS utilized to deliver various types of drugs. CS is a positively charged and linear PS consisting of *N*-acetyl-d-glucosamine (i.e., 2-acetylamino-2-deoxy-d-glucose) units linked by β-1,4 linkages. CS is produced by the deacetylation of chitin, which is the second most abundant PS on earth (Miao et al., 2018; Qu and Luo, 2020). The CS-based products have been widely used in the food and cosmetic industry as well as medicine such as wound dressing (Morin-Crini et al., 2019). In addition, the bioactivities of CS include antibacterial, antifungal, anti-HIV-1, and antioxidant activities (Cheung et al., 2015). When used in drug delivery, the CS-based NP demonstrate good biodegradability, low toxicity, and mucoadhesive ability (Lapitsky, 2014; Swierczewska et al., 2016). The CS shows poor water solubility at neutral pH, while the acidic environment (i.e., pH <6.5) and improved deacetylation can help solubilize the CS (Miao et al., 2018). Due to its primary amino groups, CS appears to be positively charged, which confers its excellent ability to bind anionic therapeutics in drug delivery (Debele et al., 2016; Ojeda-Hernandez et al., 2020). The ionic gelation of CS is one of the most attractive methods to formulate the self-assembled CS NG, which does not require chemical reactions.

**Figure 1:**
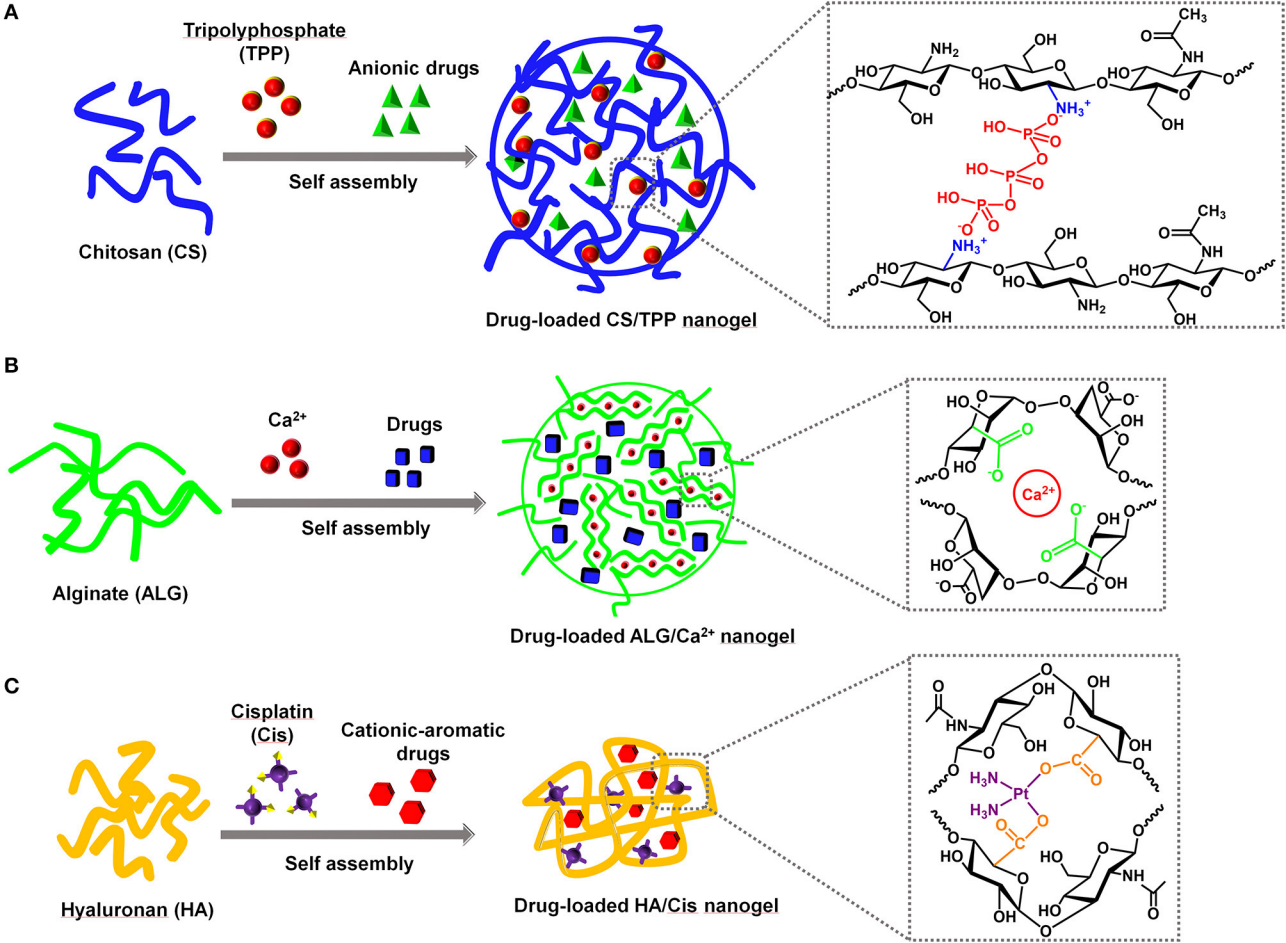
The representative self-assembled nanogels based on the natural polysaccharides (PS) for drug delivery. The order of mixing PS, drugs, and ionic cross-linkers could be varied during the self-assembly process. **(A)** CS/TPP nanogel, **(B)** ALG/Ca^2+^ nanogel, **(C)** HA/Cis nanogel.

The positive charges of CS allow it easily to interact with polyanionic molecules to form hydrogels. Tripolyphosphate (TPP) is the most widely used non-covalent cross-linker for CS due to its non-toxic and multivalent properties (Fan et al., 2012; Fischetti et al., 2020). The negative charges of TPP could efficiently interact with amines on CS through electrostatic interaction, which leads to CS cross-linking and thus the formation of NG. The CS/TPP NG was able to withhold anionic drugs through their electrostatic interaction with CS (Figure 1A). First, Calvo et al. demonstrated that the CS together with polyethylene oxide copolymer could be physically cross-linked by TPP to form NG ranging from 200 to 1,000 nm to load bovine serum albumin (Calvo et al., 1997). Currently, the typical preparation of drug-loaded CS/TPP NG only requires the mixing of CS, TPP, and the drug of interest followed by a purification procedure. During the self-assembly process, the parameters, such as the pH, temperature, concentrations of TPP and drugs, and the molecular weights of CS, are the key factors to control the physicochemical properties of the CS/TPP NG (Fan et al., 2012; Desai, 2016; Sreekumar et al., 2018). Due to the facile preparation, a quick survey of self-assembly conditions for CS/TPP NG as well as the large-scale manufacturing is possible. An increasing number of anionic drugs, such as small-molecule drugs, nucleic acids, and proteins, have been successfully encapsulated in CS/TPP NG to realize various therapeutic applications (Nogueira et al., 2013; Desai, 2016; Jamil et al., 2016). Recently, Abbaszadeh et al. reported that the CS/TPP NG loaded with quercetin could potentially function as both antibiotic and anticancer agents (Abbaszadeh et al., 2020). When compared with the small-molecule drugs, the delivery of nucleic acids, such as genes, messenger RNA (mRNAs), and small interfering RNA (siRNAs), faces more challenges due to their vulnerability to nuclease degradation and poor capability to cross the cell membrane. Besides, the endosomal escape of nucleic acids was necessary to ensure an effective transfection, since nuclear and cytoplasmic locations are essential for gene and siRNA delivery, respectively (Degors et al., 2019). The positively charged CS could efficiently bind and compact the nucleic acids, thus protecting them from nuclease degradation. Katas et al. demonstrated that, compared with the direct complexation of CS and siRNA, the CS/TPP NG mediated a better RNA interference (RNAi) effect that was presumably due to the improved binding and loading of siRNA in the NG (Katas and Alpar, 2006). On the cellular uptake of CS/TPP NG, CS can also mediate the endosomal escape of the nucleic acids, since amines of CS induce the proton sponge effect to mediate endosomal destabilization (Nasti et al., 2009; Richard et al., 2013). Similar to nucleic acid delivery, CS/TPP NG could load different proteins, protect them from enzyme degradation, and realize their cytosol delivery (Xu and Du, 2003; Renu and Renukaradhya, 2020). Although the cationic property of CS/TPP provides a flexible delivery platform for different types of drugs, it also brings unnecessary effects. In the case of protein delivery, the strong positive charges of CS may induce the conformational change of protein, which inevitably compromise the protein activity (Bekale et al., 2015; Moraru et al., 2020). Additionally, positively charged CS/TPP NG may damage the cell membrane, causing unexpected cell toxicity (Bowman and Leong, 2006). Similar to other cationic NP, CS/TPP NG also induce the non-specific adsorption of proteins from the biological fluid, which may not only change the size of NP but also affect the *in vivo* circulation (Corbo et al., 2016; Moraru et al., 2020). To reduce the positive charges of CS/TPP NG, other anionic polymers could be co-encapsulated to neutralize a part of the positive charges, thus potentially reducing the non-specific side effects.

## Self-Assembled Alginate (ALG) NG

ALG is the second most used PS to construct ionic hydrogels for different biomedical purposes. ALG is a linear anionic PS mainly derived from brown algae and bacteria, consisting of β-d-mannuronic acid (i.e., M units) and α-l-guluronic acid (i.e., G units) (Debele et al., 2016; Miao et al., 2018). ALG has been approved as food additives and widely used in biomaterials researches ascribing to its minimal toxicity, low cost, ease to formulate hydrogels, and mechanical flexibility (Lee and Mooney, 2012). The functional groups of ALG, such as hydroxyl and carboxyl groups, can non-covalently interact with divalent cations, such as Ca^2+^, Zn^2+^, and Mn^2+^ (Russo et al., 2007; Brus et al., 2017), which mediate the ionic gelation and the NG formation. The G residues of ALG show a high affinity to divalent cations such as Ca^2+^ than M residues. Consequently, ALG with more G blocks leads to hydrogels of better stability compared with ALG that is rich in M blocks (Debele et al., 2016). Nevertheless, the self-assembly of ALG mediated by the electrostatic interaction with cations enables a facile preparation of drug-loaded NG. The resultant NG features the flexibility to deliver different drugs such as chemotherapeutics for cancer, insulin for diabetes, and antibiotics for infection diseases (Severino et al., 2019).

Calcium is the most extensively used cation for ALG NG preparation since it is an essential element for the body and easily accessible. First, Grant et al. reported that the ionic interaction between G blocks and Ca^2+^ formed an “egg-box” structure and contributed to the physical cross-linking of ALG (Grant et al., 1973; Hu et al., 2021). The Ca^2+^-mediated gelation could initiate the formation of ALG/Ca^2+^ NG to encapsulate different drugs through self-assembly (Figure 1B). Under the selected preparation conditions, a direct mixing of ALG and Ca^2+^ could generate drug-loaded NG without the involvement of solvents (Xue et al., 2015; Bazban-Shotorbani et al., 2016). Xue et al. demonstrated that doxorubicin could be electrostatically self-assembled into the ALG/Ca^2+^, and thus the NG showed a pH-responsive drug release behavior, excellent compatibility, and anticancer effect (Xue et al., 2015). Generally the ALG/Ca^2+^ NG formation is mainly dependent on the non-covalent interactions between ALG, Ca^2+^ and the encapsulated drugs. The concentration of each component, the cross-linking time, and the pH to prepare NG could control the final parameters of ALG/Ca^2+^ NG such as the size, drug encapsulation efficiency, and drug release behavior (Choukaife et al., 2020). Despite the facile preparation and promising biomedical applications of the self-assembled ALG NG, a comprehensive evaluation of the biocompatibility of ALG is also required for the applications of drug delivery. Most of the ALGs are extracted from algae and bacteria, and meanwhile the preparation of ALG NG may require the assistance of solvent. The impurities, such as endotoxin and residual solvent in ALG NG, could also induce unexpected side effects. Endotoxins are reported to induce immunogenicity and thus compromise the biocompatibility of ALG NG (Lee and Mooney, 2012; Choukaife et al., 2020). The direct self-assembly of purified ALG and drug without the involvement of strong solvent may represent a potent strategy for drug delivery with improved safety profiles.

## Self-Assembled Hyaluronan (HA) NG

Besides the CS and ALG, the reports of HA for PS NG preparation are increasing quickly for the past decade. HA, also named as hyaluronic acid and hyaluronate, is a linear and non-sulfated PS composed of repeating disaccharides of d-glucuronic acid and *N*-acetyl-d-glucosamine units (Jeong et al., 2008). HA is highly negatively charged and a major component of the extracellular matrix of mammalian cells (Debele et al., 2016; Miao et al., 2018; Yu et al., 2020). Due to its excellent biocompatibility, low cost, non-immunogenicity, and water-binding properties, HA has been used for dermal filler, joint lubrication, prevention of surgical adhesion, dietary supplements, and eye drops (Valachova et al., 2016; Kim et al., 2017). HA could be specifically recognized by its receptors such as CD44, endocytosed and trafficked to endolysosomes, degraded by hyaluronidase, and then recycled for HA synthesis (Gao et al., 2021). Consequently, the HA-based drug delivery system could realize CD44-targeting as well as hyaluronidase-responsive drug delivery for different cancers. The carboxylic acid, *N*-acetyl groups, and primary and secondary hydroxyl groups on HA provide chemical modification sites to produce numerous HA derivatives, such as HA-drug conjugates, which have entered the clinical trials (Miao et al., 2018). The carboxyl groups of HA also provide non-covalent cross-linking sites for both Fe^3+^ and cisplatin to generate NG through self-assembly (Jeong et al., 2008; Isayeva et al., 2010; Tian et al., 2016; Zhang and Tung, 2017).

Currently, the most used non-covalent cross-linker for HA NG is the cisplatin. Cisplatin is a first-line chemotherapeutics for multiple cancers. After the cellular uptake, cisplatin could be aquated and thus get activated in the cytosol (Johnstone et al., 2016). The anticancer activity of cisplatin relies on the DNA cross-linking mediated by the platinum coordination. Similar to its coordination with DNA, cisplatin could also coordinate with the carboxyl groups of various polymers, e.g., HA (Uchino et al., 2005; Jeong et al., 2008; Zhang et al., 2017). The early preparation of HA and cisplatin (HA/Cis) NG employs the silver nitrate to induce the cisplatin aquation, which facilitates its coordination with HA (Cai et al., 2008; Jeong et al., 2008). A simple heating of HA and cisplatin was then found to be capable of accelerating the self-assembly process and thus efficiently tuning the size of the resultant NG (Li and Howell, 2010). The cisplatin in HA/Cis NG not only functions as a cross-linker during the NG formation but also acts as a drug on its release. The existence of chloride reverses the coordination between cisplatin and HA, which could realize a controlled release of cisplatin and thus reduced its notorious side effects in cancer chemotherapy (Cai et al., 2010; Ishiguro et al., 2016). Interestingly, HA and cisplatin could also be co-assembled with a second drug to form multidrug NG (Figure 1C), thus easily realizing the combination therapy especially for cancer (Zhang and Tung, 2017). The cationic-aromatic drugs (CA drugs) containing aromatic ring and positive charges, such as chemotherapeutics (Zhang et al., 2017, 2018; Yu et al., 2020; Ma et al., 2021), kinase inhibitors (Zhang and Tung, 2017), and photosensitizers/dyes (Zhang and Tung, 2018a), could be easily self-assembled into the HA/Cis NG. The positive charges of CA drugs are believed to interact with HA through electrostatic interaction while the aromatic ring contributes to the hydrophobic interaction and π-π stacking of drugs, which collectively facilitates the encapsulation of CA drug into the NG. Typically, heating of the mixture of HA, cisplatin, and CA drugs could produce a multidrug HA NG. The heating time, ratio of cisplatin to CA drug, and the CA drug identity were found to determine the final physicochemical properties of the NG such as size, the encapsulation efficiency of drug, and the release speed of drug (Zhang and Tung, 2017). Currently, doxorubicin, gefitinib, dasatinib, toluidine blue, etc. have been successfully co-assembled into the HA/Cis NG (Gao et al., 2021). The HA/Cis NG with doxorubicin encapsulation demonstrated a pH- and GSH-responsive release behavior, which was able to help combat the cancer drug resistance (Zhang and Tung, 2018b; Ma et al., 2021). Generally, the HA/Cis NG could efficiently realize a CD44-targeted delivery for cancers such as cisplatin monotherapy and cisplatin-based combination therapy. However, cisplatin as a cross-linker in HA/Cis NG inevitably limits its application to malignant diseases. After the self-assembly of HA, cisplatin and CA drugs, a simple dialysis in phosphate-buffered saline (PBS) could remove the cisplatin while keep the CA drugs retained in the NG (Zhang et al., 2017). Subsequently, cisplatin could also be a removable cross-linker to help construct self-assembled HA NG loaded with a single drug, which potentially extends the application of HA NG to other non-cancerous diseases. Different CA drugs could be self-assembled into the HA/Cis NG, and it is still a challenge to realize the macromolecular drug delivery using the native HA.

## Conclusions and Perspectives

The ionic gelation mediated by electrostatic interaction and metal coordination has provided a very facile and biocompatible option to construct the drug-loaded NG through the self-assembly of natural PS such as CS, ALG, and HA. Other than the three PS discussed earlier, ionic gelation of gellan gum and pectin with cationic ions were also used; however, the applications of these PS NG were relatively rare (Racovita et al., 2009; Pedroso-Santana and Fleitas-Salazar, 2020). Future efforts can also focus on exploring different PS NG using the self-assembling strategy for drug delivery. A full understanding of the mechanism between the self-assembly of native PS, ionic, or metal cross-linkers as well as the drug of interest will help quickly obtain the drug-loaded NG with desired properties. For example, the gelation of CS by the TPP was occurred instantly, which made the mechanism study of the NG formation more challenging (Desai, 2016). Adding sodium chloride during the CS/TPP NG preparation could screen the charges and slow the gelation process, which also helped narrow the NG distribution with improved stability (Huang and Lapitsky, 2011, 2017). In terms of the application of drug delivery, ideally, the drug needs to be specifically delivered to the site of action while reducing its non-specific toxicity as much as possible, which requires the nanocarriers to overcome different barriers in the body (Polo et al., 2017; Mitchell et al., 2021). The self-assembled PS NG provide multiple benefits to overcome a part of the above barriers. However, disadvantages also existed for each specific PS NG in the drug delivery. The hydrophilic HA itself has an antifouling property, and meanwhile, it could mediate a targeted delivery of drugs to the tumor with CD44 overexpression (Xia et al., 2019; Lee et al., 2020). However, most of the HA NP are retained in the endolysosomes following the trafficking of HA, which largely prevents the delivered drugs to access their molecular targets and subsequently limits the final therapeutic efficacy. To increase the cytosolic drug delivery of HA NG, the improvement in their lysosomal escape capability may also be considered. Currently, several applications of PS-based drug delivery are in different phases of clinical trials (Miao et al., 2018). Although most of them are PS-drug conjugates, the successful demonstration of these PS-based formulations in patients strongly supports the promising therapeutic benefits of PS as a drug carrier. For example, the self-assembled HA/Cis NG (HylaPlat) is being evaluated in phase I/II clinical trial for cancers in dogs (Cai et al., 2016). Generally, the self-assembled NG using the native PS that are free of chemical reactions and meanwhile with ease to scale up will have their own advantages for the translational applications. Before their clinical translation, a thorough evaluation of the therapeutic efficacy as well as the comprehensive safety evaluation are highly recommended using the clinically relevant animal models according to the independent application.

## Author Contributions

WZ and HW conceived and drafted the manuscript including the figures. HD and MG participated in reviewing the literature, writing, and discussions. All authors approved the submitted version.

## Conflict of Interest

The authors declare that the research was conducted in the absence of any commercial or financial relationships that could be construed as a potential conflict of interest.
